# Subjective Quality Assessment of Underwater Video for Scientific Applications

**DOI:** 10.3390/s151229882

**Published:** 2015-12-15

**Authors:** José-Miguel Moreno-Roldán, Miguel-Ángel Luque-Nieto, Javier Poncela, Víctor Díaz-del-Río, Pablo Otero

**Affiliations:** 1 Department of Communication Engineering, University of Málaga, 29071 Málaga, Spain; luquen@uma.es (M.-Á.L.-N.); jponcela@uma.es (J.P.); pablo.otero@uma.es (P.O.); 2 Spanish Institute of Oceanography, 29649 Fuengirola, Spain; diazdelrio@ma.ieo.es

**Keywords:** video quality, MOS, quality assessment, underwater sensor networks

## Abstract

Underwater video services could be a key application in the better scientific knowledge of the vast oceanic resources in our planet. However, limitations in the capacity of current available technology for underwater networks (UWSNs) raise the question of the feasibility of these services. When transmitting video, the main constraints are the limited bandwidth and the high propagation delays. At the same time the service performance depends on the needs of the target group. This paper considers the problems of estimations for the Mean Opinion Score (a standard quality measure) in UWSNs based on objective methods and addresses the topic of quality assessment in potential underwater video services from a subjective point of view. The experimental design and the results of a test planned according standardized psychometric methods are presented. The subjects used in the quality assessment test were ocean scientists. Video sequences were recorded in actual exploration expeditions and were processed to simulate conditions similar to those that might be found in UWSNs. Our experimental results show how videos are considered to be useful for scientific purposes even in very low bitrate conditions.

## 1. Introduction

Underwater imagery is an essential tool in the research and study of the oceans since it provides invaluable information about the mostly unknown contents of the seabed and the water column. Beyond the scientific utility, other applications in the fields of defense, tourism or fishery can also be envisioned. The gathering of scientific underwater video footage is currently very expensive since it involves exploration expeditions with vessels in which divers (shallow waters) or robots are submerged for limited recording sessions.

In this context, the deployment of wireless sensor networks capable of video capturing and transmission would be a major technological breakthrough allowing for continuous monitoring or cooperative exploration with autonomous underwater vehicles (AUVs). A video service with enough quality could lead to decisions about the re-planning of AUVs path even if instant remote controlling were not possible due to transmission delays. However, current underwater wireless networks experience limited capabilities which brings into question the feasibility of video services. The analysis of the perceived quality constitutes a very important aspect for service provisioning since it reveals which quality could be achieved under certain conditions. Moreover, if some of the attainable qualities are enough for the target applications intended by ocean scientists. Our initial hypothesis is that ocean scientists’ opinion will be influenced by the comparative advantages of the service: the possibility of a continuously available source of images and a vastly reduced delay, in spite of the limitations of the network capabilities and their impact in the achievable quality.

Section 1.1 of this paper is a survey of the state of the art in the subject of video quality assessment. Section 1.2 briefly introduces the standard methodologies for quality assessment and their suitability for the environment under study. Section 2 thoroughly describes the experiment conducted to obtain quality assessment data. Section 3 presents the results of the experiment. Section 4 contains the conclusions drawn from these results.

### 1.1. Related Work

Multimedia data acquisition is currently difficult in wireless underwater networks due to the low data rates available. Proposals in existing literature try to circumvent this obstacle with different solutions. In [1], pictures captured by sensor nodes are gathered by an autonomous underwater vehicle (AUV). This AUV travels to the position of each node and downloads the information through an optical link. This method suffers from delays due to the time required to complete a round trip through all deployed sensors. [2] suggests a different setting with underwater sensors wired to a buoy equipped with a communication unit transmitting over the air with an 802.11b modem. This kind of dual node requires heavy anchoring, is only suitable for shallow waters and is more vulnerable to potential damages. Other existing studies include image quality considerations. [3] proposes three classes of quality of service (QoS) to optimize the network performance and acknowledges that mechanisms to meet application level QoS requirements and to map them into network-layer metrics have not been primary concerns in mainstream research on UWSN. Although the authors make a significant contribution with their cross layer protocol, assessing the quality remains an unaddressed task. [4] does obtain a quality measure of the studied service but it only considers still images and the quality is assessed through computing peak signal to noise ratio on standard test images (not even related to the underwater context). This is an objective method which does not provide direct information about how these images will be rated by the users of the service or how useful they will be for them.

The present paper is motivated by the need to have subjective QoS data for video services under the constraints of the underwater medium. The importance of subjective quality assessment is supported by similar studies already performed such as [5] for general purpose video, [6] for mobile video or [7] for http based streaming. As a result of these three papers, a remarkable database with subjective quality information is publicly available [8]. However, all of these works use videos with much higher bitrates than those achievable in UWSNs. Some previous works highlight the effect of network parameters such as the packet loss rate in [9]. Other works highlight environmental viewing conditions as the relation between the viewing distance and the image resolution [10]. Studies on how a specific service and their users can affect the quality assessment process can also be found in [11] for telemedicine multimedia applications. In the latter, the conclusions emphasize the differences in quality perception when the video application is being used by a medical expert and how this is highly dependent on the specialty area.

Other papers on this topic focus on general models for opinion-based quality estimation. A thorough compilation and comparison can be found in [12] along with the authors’ own proposal. The reference model is [13], which is part of the International Telecommunication Union (ITU) standard “G.1070 Opinion model for video-telephony applications” [14]. Although targeted for a very particular application, it has been used for other multimedia services due to the lack of a more appropriate standard for video quality assessment. Other models cited in [12] differ from Yamagishi’s proposal in the video and network parameters that they take as input variables (e.g., bitrate, frame rate, packet loss, video content). Conclusions of [12] show that the best estimations are computed with their model and the G.1070 model, depending on the video impairment type, but the performance analysis is made with bitrates that cannot be attained in UWSNs. Nevertheless, all the analyzed models share the main limitation that a set of coefficients derived from subjective data is needed to complete the model equations. These coefficients are linked to certain input variable ranges and some additional settings like video size, resolution or compression codec. The models only provide sets of coefficients for a small group of settings. Outside these configurations they cannot be used unless new coefficients are computed. For example, three out of five sets of coefficients included in G.1070 require video bitrates over 256 kbps, a figure far from the achievable rate with current acoustic technology. The above-mentioned drawbacks of parametric models make subjective quality assessment essential in the feasibility analysis of multimedia services for UWSNs. The present paper focuses in gathering this kind of data for underwater networks conditions, which is, to the best of our knowledge, missing from the current available bibliography.

### 1.2. Quality Assessment Methods Overview

According to existing standards and literature, quality for a network service can be assessed with two different kinds of approaches: subjective methods and objective methods. Both approaches usually aim to produce a standard quality metric known as Mean Opinion Score (MOS) which represents observed (for subjective methods) or estimated (for objective methods) quality in a numeric scale. The standard range is [1, 5] (higher is better) although some other scales can be used for particular applications ([1, 10] interval is used for a higher discriminant capability). The first group of techniques aims to get quality values directly from human evaluators. A group of viewers (in the case of video services) are presented a sequence of stimuli which they are asked to score on a quality scale. These scores are statistically processed to compute MOS values for different conditions of service provisioning. The second group of methods pursues the estimation of MOS values from a mathematical model. Objective methods are classified by ITU [14] in three categories according to the inputs they take:

1. Intrusive monitoring: both transmitted and received signals are required as inputs.
2. Non-intrusive monitoring: only the received signal is used as input.
3. Network planning: no signal needed, estimation is based on network parameters for the transmission.

Subjective methods have a clear disadvantage in terms of the amount of resources needed, since time and people are required. Objective methods are faster and only necessitate an implementation of the algorithm for computing the MOS. The parametric scheme seems the most suitable option for the current scenario.

**Figure 1:**
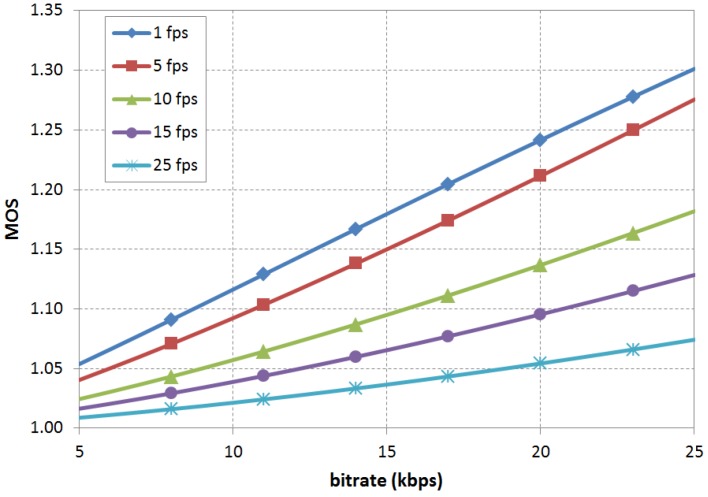
MOS values predicted by G.1070 for MPEG4, QVGA and 4.2’’ videos.

The ITU G.1070 model is here taken as a reference since it is the only standardized one. Five coefficient groups are provided (as non-normative values); two of them have no constraints regarding bitrate range. However, full details for scene characterization, used for source subjective values, cannot be found in bibliographic references. Provided examples include no bitrates under 32 kbps, but this is the range under study due to technological limitations (see Section 2.1). Figure 1 shows the MOS values predicted by this model for a 5–25 kbps bitrate interval. Different curves are plotted for common frame rates and the coefficient group corresponds to MPEG4, QVGA and 4.2’’ sized images. The highest MOS is predicted for 25 kbps and 1 fps with a value of 1.301 in a one to five scale, low enough to question the utility of this kind of video.

An additional problem found in current parametric video quality models is the lack of an “advantage factor”. This factor characterizes the fact that users give higher scores if the service allows communication in situations with special access conditions. It is defined in the ITU standard for audio quality G.107 [15], which is used for the audio quality estimation in G.1070. This compensation could be particularly important for underwater video context in which image acquisition is challenging as explained before.

Video quality assessment through objective methods is difficult in UWSNs since monitoring is virtually unavailable and parametric estimation reliability needs to be checked. Therefore, a subjective study seems the most appropriate way to tackle the quality measurement task. The ITU standardized process specified in BT.500 [16] recommendation has been used for a long time although it is intended for television pictures. A more recent standard for “Subjective video quality assessment methods for multimedia applications” is described in P.910 [17]. Both recommendations are quite similar in the main aspects. Following this later recommendation, an experiment to determine video quality under conditions of interest was conducted. The details about the experiment design and setup can be found in the next section.

## 2. Experiment for Subjective Quality Assessment 

Subjective quality assessment in video services requires a careful planning accounting for all the different aspects of the experiment. The specific features of the target service in terms of video configuration parameters are detailed in Section 2.1. The following experimental set-up information, as described in P.910 recommendation, is provided: details about the recording environment and equipment producing the source signal (Section 2.2); scene creation and selection criteria (Section 2.3); psychometric method used in the quality test (Section 2.4); equipment and settings used for performing this test (Section 2.5); full description of the viewing conditions (Section 2.6).

### 2.1. Target Service

Video services studied here could be provided in wireless sensor networks with anchored nodes for monitoring underwater ecosystems or with autonomous vehicles for visual exploration of seafloors. Current underwater networks are in an early stage of development and state of the art acoustic modems reach a peak data rate of 62.5 kbps with a 300 m operating range [18]. Bitrates available in the application layer are highly limited and the quality is seriously burdened by this constraint.

Nevertheless, underwater video as considered in this paper is not a service to be provided to a great number of heterogeneous users like other video services such as IPTV, videoconferencing or video-sharing streaming. Instead, it is considered as a tool for a very specific public and a particular use: scientific exploration and monitoring of areas that are otherwise very difficult and expensive to reach. We also need to consider the specific features of state-of-the-art differential video encoders which leverage intra-frame and inter-frame similarities. Because of this, a direct relation between parameters cannot be found, *i.e*., a 10 fps video is not twice the size of the 5 fps equivalent.

Taking this into account, a sensible choice of video parameters (the quality of which will be assessed in the experiment) has been based on a previous study [19]. In this preliminary analysis a simplified quality test was conducted among a reduced group of viewers (not related to ocean science) leading to the following choice for the current experiment:

○ Bitrates: 8, 14, 20 kbps.
○ Frame rates: 1, 5, 10 fps.
○ Resolution: 320 × 240, 160 × 120 pixels.
○ Color depth: RGB (3 × 8 bits) and Grayscale (8 bits) video.

In this previous work, the bitrate was found to be the main limiting factor. Below 8 kbps, video contents were difficult to distinguish, even in low resolutions. An effective data rate higher than 20 kbps is unrealistic due to protocol overheads and competition among several nodes. In these bitrate conditions, a standard 25 fps rate produced fuzzy images. For equivalent conditions, viewers preferred videos with lower smoothness with enhanced frame sharpness. This fact agrees with the behavior predicted by G.1070 model, which suggests that below a bitrate threshold, lower frame rates offer better quality. However, for two MOS vs bitrate curves plotted for different frame rates, the bitrate value for the crossing point depends on the particular frame rates being compared. Because of this, the selection of frame rates for the current experiment ranges from 1 to 10 fps.

Concerning image size, only low resolutions (QVGA, QQVGA) are suitable for these conditions since higher resolution pictures would appear hazy and suffer from artifacts. Finally, the impact of color/grayscale streams in quality will also be studied.

### 2.2. Recording Environment and Source Signal

Video sources for this experiment have been provided by the Spanish Institute of Oceanography (Instituto Español de Oceanografía, IEO) from real exploration expeditions in the scope of the project “Life+Indemares-Chimeneas de Cádiz” [20]. Images were captured with the underwater vehicle VOR APHIA 2012, a prototype developed by the GEMAR research group (IEO). It includes its own illumination system consisting of two high power LED spotlights of 19,000 lumens in a 60° angle and a Canon Legria HF R106 as camera/recording system. This camcorder features a 1/5.5 type CMOS sensor and AVCHD as video encoding format with 4:2:0 color sampling scheme. Recording settings are 1440 × 1080 pixels resolution, 25 frames per second, automatic white-balance and automatic focus. AVCHD is a compressed format using H.264. The quality provided is considered to be high enough so that there are no visible compression artifacts, particularly considering that the test sequences will be downscaled by a 4.5 linear factor.

### 2.3. Scene Selection

Image contents range from almost static shoots of plain sea bottom to fast navigation of areas with complex layouts of irregular rocks and different kinds of underwater flora and fauna. As a representative sample of these conditions, a number of 56 scenes with a 12 s duration were chosen to be used as potential test sequences. All the video manipulations needed for the test have been made with Avidemux 2.6 software. This includes clipping, resampling, downscaling and re-encoding.

H.264 differential compression produces a variable bitrate video. The average bitrate of a time window within the scene does not have to match the average bitrate for the whole scene. The bitrate for a set of frames depends on the previous frames, their bitrate and the adjusting bitrate algorithm. Every 12 s sequence is encoded at a random starting position within a longer 120 s clip. This way, we randomize the unwanted effects due to the variable bitrate coding. The 120 s segments have been processed to generate test sequences with all possible combinations of parameters mentioned in Section 2.1.

Two important attributes for scenes are the spatial and temporal perceptual information because high variation scenes will be further impaired when encoded. To measure the characteristics of the scenes in these two dimensions we have generated sequences with the largest evaluation resolution (320 × 240 pixels) and a very high bitrate (3000 kbps). This bitrate guarantees that there are no impairments in the clips aside from those due to the reduced resolution. The metrics for spatial perceptual information (SI) and temporal perceptual information (TI) are specified in Equations (1) and (2) [17]. These metrics, as defined in the recommendation, are only applied to the luminance plane of the images (*F_n_* is the *n*-th frame represented as a pixel matrix containing this luminance information). The Sobel operator in Equation (1) is a convolutional kernel operator used for edge detection [21], the result of applying this operator over a frame is also a pixel matrix. The std_space_ operator computes standard deviation of luminance values within a single pixel matrix. The max_time_ operator selects the maximum value of the argument (spatial standard deviation for a pixel matrix in both cases) over the set of all processed video frames in the clip:

(1) $$\text{SI}={\text{max}}_{time}\left\{{\text{std}}_{space}\left[\text{Sobel}\left(F_{n}\right)\right]\right\}$$

(2) $$\text{TI}={\text{max}}_{time}\left\{{\text{std}}_{space}\left[M_{n}\left(i,j\right)\right]\right\},\text{ with }M_{n}\left(i,j\right)=F_{n}\left(i,j\right)-F_{n-1}\left(i,j\right)$$

**Figure 2:**
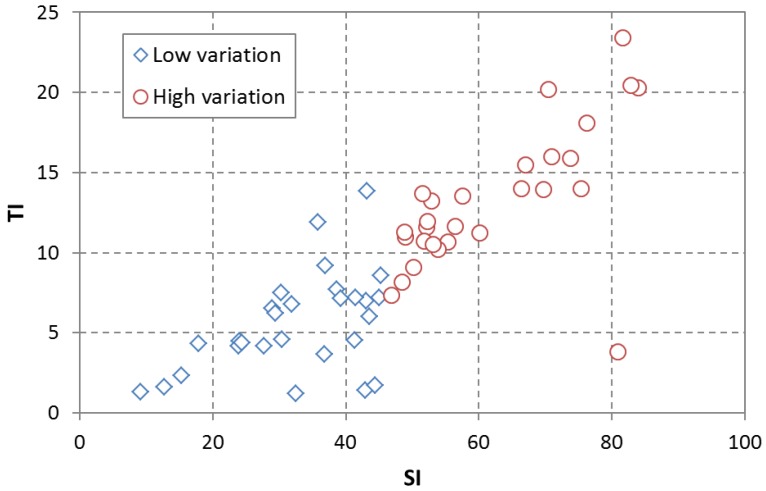
Scatter diagram for Spatial and Perceptual Information in test scenes.

Figure 2 shows the (SI, TI) plane for all sequences. It can be observed that no samples but one have a high value for one dimension and a low value for the other dimension. The Pearson correlation coefficient for both dimensions is ρ = 0.8416, which shows a substantial linear dependence. Scenes have been divided in two groups according to their content variation features. The threshold is the median in the variable with the largest scattering, SI.

Scenes in the same group are considered equivalent in terms of content variability. Instead of choosing a few representative scenes, repeated with every configuration, each evaluation condition is applied to a different video content. Thereby we avoid two important side effects observed in the previous study [19]:

1. Learning effect: viewers can recognize objects from a low quality scene if they have seen them previously in a better quality. Thus, their opinion could be biased.
2. Boredom effect: even in short sessions (less than fifteen minutes) volunteer viewers get bored if the same contents are displayed repeatedly. This may cause a loss of interest and introduce unwanted factors in the test.

Initially, contents from the 56-sample scene database were intended to be randomly assigned to each viewing condition according to content variation required (see Section 2.6). However, a close inspection of the clip file sizes showed that the average bitrate defined in the compression settings was sometimes ignored. Compressed scenes with a deviation higher than ±10% of the target size were removed from the database and only the remaining clips were employed in the random assignment.

### 2.4. Test Method

The absolute category rating (ACR) method described in P.910 has been used to assess video quality. It employs a standard five-level scale: bad, poor, fair, good and excellent. This method requires a short explanation time and the single stimulus presentation is the most similar one to the typical use of the video sequences.

**Figure 3:**
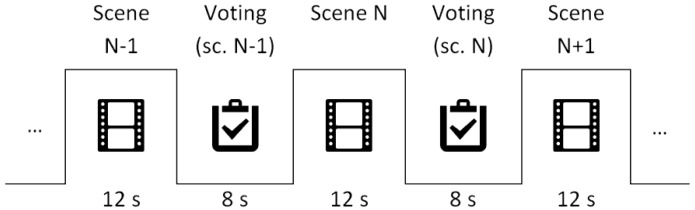
Scene-voting sequence time pattern.

Some viewers can feel unsure how to use the scale and change their evaluation criteria after some scenes. Taking this into account, some “dummy” scenes have been introduced at the beginning of the test to stabilize viewers’ ratings. Figure 3 shows the time pattern for the presentation: 12 s for scene visualization and 8 s for voting.

Once the viewers had ended the scene scoring, they were asked to rate the scientific utility of the quality categories they had just used in the test. They were provided a five rank (ACR-like) scale with the following values: useless, barely useful, moderately useful, quite useful and very useful. This question was designed to go beyond the perceived quality as an abstract concept, linking it to another magnitude, also subjective but with a more specific meaning. After delivering the test form viewers went through a short interview in which they were asked to give a qualitative opinion about the images they have just seen.

### 2.5. Evaluation Procedures

The playback system was an HTML5 application developed specifically for this purpose. The application starts with an instruction screen with a start button. Once the button is pressed, clips are presented in sequence (see Figure 3) and no further interaction is required from the user. Viewers were informed about the test procedures and given a paper form to write down the score for each scene. The application was displayed in a 14’’ screen configured with WXGA (1366 × 768 pixels) resolution. Scenes were centered in the screen with visualization size 320 × 240 pixels (diagonal length 3.57’’) for both resolutions with the background color set to 50% grey.

Illumination conditions were measured using a photometer (Sekonik L-758DR [22]) for both the screen and the room where the test was conducted. Table 1 collects the illumination requirements given in recommendation P.910 alongside with the measured values in the test.

**Table 1:** Illumination conditions.

Parameter	Requirement	Measured Value
Peak luminance of the screen.	100–200 cd/m²	111.4 cd/m²
Ratio of luminance of inactive screen to peak luminance.	≤0.05	0.001
Ratio of luminance of the screen when displaying only black level in a completely dark room to that corresponding peak white.	≤0.1	0.004
Ratio of luminance of background behind picture monitor to peak luminance of picture.	≤0.2	0.006
Background room illumination	≤20 lux	2.5 lux

Chromaticity was not measured since required D65 illuminant corresponds with daylight and the only source of light in the room was natural light shaded by adjustable panels. Illumination conditions were kept constant during the entire test.

The viewing distance was approximately 50 cm or 8 H using the picture height as reference (as defined in P.910). This matches the conditions in which the images would be used in a real service (an application on an office computer).

A minimum of four viewers are required for statistical processing, although P.910 suggests at least 15 viewers should participate in the experiment. They should not be directly involved in picture quality evaluation. A total of 21 viewers took part in the test, all of them ocean scientists, geologists and biologists, from the Oceanographic Málaga Center of the IEO. The group features a wide variety of research interests such as sedimentology, submarine morphology, plankton, taxonomy of small species, benthos, and fishery. Some of them were acquainted with the use of images in their everyday work although none of them had been involved in video quality assessment before. Therefore, this sample of viewers met the requirements.

### 2.6. Test Conditions

Each evaluation condition is a combination of test variables which characterizes a scene to be scored by viewers. The test consisted of 31 evaluation conditions arranged in five blocks as follows:

○ Block 1: dummy conditions for score stabilization (see Section 2.4). The opinions issued for these conditions were discarded and not included in the test results.
○ Block 2: conditions for measuring the impact of the three levels of bitrate and frame rate with low variation content. Resolution and color settings for this block are QVGA and RGB.
○ Block 3: conditions for measuring the impact of the two levels of resolution and color with low variation content. Bitrate and frame rate settings for this block are 20 kbps and 5 fps.
○ Block 4: conditions for measuring the impact of the three levels of bitrate and frame rate with high variation content. Resolution and color settings for this block are QVGA and RGB.
○ Block 5: conditions for measuring the impact of the two levels of resolution and color with high variation content. Bitrate and frame rate settings for this block are 20 kbps, 5 fps.

In this block set up, only two parameters are changing within a block. This configuration allows for a better statistical analysis of results (see Section 3) which would be otherwise difficult to interpret.

Blocks are presented in the same order to all viewers but scenes within a block are randomly reordered for each test. This approach reduces the negative effects of specific ordering of scenes.

**Table 2:** Evaluation conditions.

Block	ID	Br ^a^	Fr ^b^	Resolution	Color	CV ^c^
1	D1	8	10	QVGA	RGB	Low
	D2	14	5	QVGA	Grayscale	Low
	D3	20	1	QVGA	Grayscale	Low
	D4	14	1	QQVGA	RGB	High
	D5	8	5	QVGA	RGB	High
2	01	8	1	QVGA	RGB	Low
	02	8	5			
	03	8	10			
	04	14	1			
	05	14	5			
	06	14	10			
	07	20	1			
	08	20	5			
	09	20	10			
3	10	20	5	QQVGA	RGB	Low
	11			QQVGA	Grayscale	
	12			QVGA	RGB	
	13			QVGA	Grayscale	
4	14	8	1	QVGA	RGB	High
	15	8	5			
	16	8	10			
	17	14	1			
	18	14	5			
	19	14	10			
	20	20	1			
	21	20	5			
	22	20	10			
5	23	20	5	QQVGA	RGB	High
	24			QQVGA	Grayscale	
	25			QVGA	RGB	
	26			QVGA	Grayscale	

^a^ Bitrate; ^b^ Frame rate; ^c^ Content Variation.

**Figure 4:**
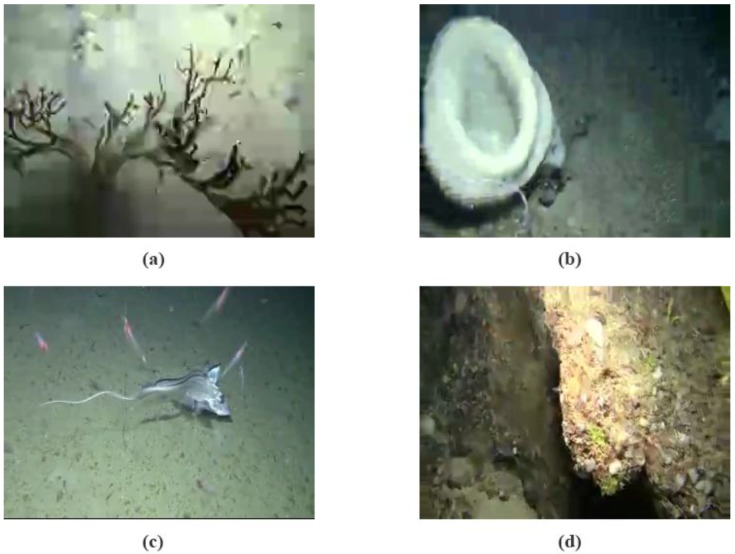
Sample frames (QVGA, RGB color). (**a**) 8 kbps–5 fps—high variation content; (**b**) 14 kbps–5 fps—high variation content; (**c**) 20 kbps–5 fps—low variation content, (**d**) 14 kbps–1 fps—high variation content.

Full detailed list of evaluation conditions is provided in Table 2. Values in combined cells are set for the whole block. Four representative frames are provided in Figure 4 as a reference for the kind of content and quality being assessed in the test.

## 3. Results and Discussion

This section describes the statistical processing of the data acquired in the experiment according to the guidelines given in P.910. The main statistic is the MOS, but other meaningful indicators have been also computed. Additionally, analysis of variance (ANOVA) tests have been performed to check the significance of the MOS differences across different blocks. 

**Figure 5:**
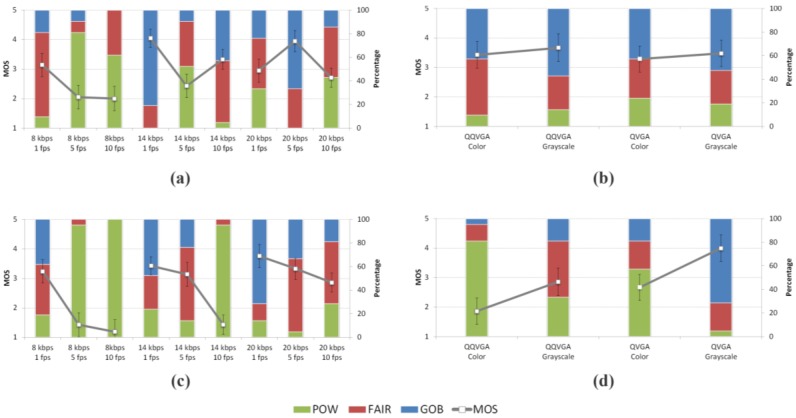
MOS values and cumulative distribution of scores as the percentage value of “good or better” (GOB-blue), fair (FAIR-red) and “poor or worse” (POW-green) scores. (**a**) Block 2; (**b**) Block 3; (**c**) Block 4; (**d**) Block 5.

Figure 5 shows, for every condition in the test, the MOS value with the 95% confidence interval (error bars). The stacked columns chart shows the cumulative distribution of quality scores in three groups of categories (each of them with a different color): the percentage of good or better (GOB) samples in the blue (upper) column, the percentage of fair (FAIR) samples in the red (middle) column and the percentage of poor or worse (POW) samples in the green (lower) column. 

Figure 5a and c plot the MOS values for low and high variation samples in the bitrate/frame rate groups (blocks 2 and 4). Apart from some exceptions it can be said that for a given bitrate, lower frame rates achieve better quality while for a given frame rate, higher bitrates are scored better. It can also be seen that the scenes with low variation contents get better scores than the high variation ones.

Figure 5b and d show the MOS values for resolution/color samples (blocks 3 and 5). Regardless of the content variation, grayscale scenes (second and fourth columns) score better than color scenes (first and third columns) for a given resolution. If color is the given parameter, higher resolutions (third and fourth columns) get a higher MOS for high variation contents while the opposite happens for low variation scenes, although in this case MOS differences are very small. As before, most of the conditions in the low variation block have better average scores than the equivalent conditions in the high variation block.

To test the statistical significance of the MOS values obtained in the tests we have used analysis of variance techniques (ANOVA) [23]. A two-way within-subjects test has been performed for each block. Using this test, we can accept or reject the hypothesis of equal means in ANOVA for each block, *i.e.*, we can attribute the differences between MOS values to changes in parameters (if we reject the hypothesis) or to other random effects in the sampling process (if we accept it). This acceptance or rejection is based on one of the results of the ANOVA test, the *p*-value, which represents a probability. A high *p*-value means the hypothesis under test may be accepted, while a low *p*-value means we should reject the hypothesis. In this experiment, two factors for each block have been defined (see Section 2.6). Three levels per factor are tested for bitrate (8, 14, 20 kbps) and frame rate (1, 5, 10 fps), while two levels are used for resolution (160 × 120, 320 × 240 pixels) and color (RGB, Grayscale). The software IBM SPSS Statistics 22 has been used [24]. We have used a significance α = 0.05 and the repeated measures option since every subject has been used to evaluate every condition in test and thus the answers for the same subject are not independent. This fact also provides improved statistical power.

The repeated measures test requires the assumption of sphericity, which is checked with the Mauchly’s test [25]. Sphericity assumption will be rejected if the result (*p*-value) of the Mauchly’s test is below 0.05. In this case, corrected *p*-values (Lower-bound, Greenhouse-Geisser [26] and Huynh-Fedt [27]) for ANOVA should be calculated. Multivariate tests do not require sphericity and are also a common tool to complete the comparison. 

**Table 3:** ANOVA results.

**Block 2—Maulchly’s Test of Sphericity**					
Within subjects effect	Maulchly’s W				Sig.
Bitrate	0.832				0.173
Frame rate	0.944				0.947
B*Fr	0.282				0.006
Block 2—Test of within subjects effects					
Source		df	MS	F	Sig.
Bitrate	S.A. ^a^	2	14.926	28.380	0.000
Frame rate	S.A.	2	8.720	19.988	0.000
B*Fr	G-G ^b^	2.705	16.967	23.405	0.000
Block 2—Multivariate tests					
Effect		Value		F	Sig.
B*Fr	Pillai’s T.	0.771		14.320	0.000
	Hotelling’s T.	3.370		13.320	0.000
**Block 4—Maulchly’s Test of Sphericity**					
Within subjects effect	Maulchly’s W				Sig.
Bitrate	0.941				0.562
Frame rate	0.853				0.221
B*Fr	0.739				0.782
Block 4—Test of within subjects effects					
Source		df	MS	F	Sig.
Bitrate	S.A.	2	29.370	69.858	0.000
Frame rate	S.A.	2	42.926	68.580	0.000
B*Fr	S.A.	4	6.140	21.158	0.000
Block 3—Test of within subjects effects					
Source		df	MS	F	Sig.
Color	S.A.	1	0.583	1.429	0.246
Resolution	S.A.	1	0.964	2.477	1.131
R*C	S.A.	1	0.012	0.041	0.841
Block 5—Test of within subjects effects					
Source		df	MS	F	Sig.
Color	S.A.	1	20.012	64.160	0.000
Resolution	S.A.	1	28.583	65.962	0.000
R*C	S.A.	1	0.583	1.522	0.232

^a^ Sphericity Assumed; ^b^ Greenhouse-Geisser.

A summarized version of the full analysis output is shown in Table 3. The table contains the main results of the analysis: sum of squares, degrees of freedom, mean squares, F ratio and *p*-value (under the “Sig.” column). For blocks testing bitrate and frame rate, the Mauchly’s sphericity test results are shown first. The hypothesis of sphericity should only be rejected for the interaction effect in block 2 (*p* = 0.006). The corrected value for significance, calculated using the Greenhouse-Geisser method, is shown in the within subjects effects table. The results of two multivariate tests (Pillai’s and Hotelling’s Traces) are also included to complete the comparison. Other multivariate tests offered by SPSS had consistent values. The computed significances allow rejecting the hypothesis of equal means for blocks 2 and 4 (*p* < 0.001) and state that changes in the MOS are due to changes in parameters. We can also say that changes for different levels of bitrate depend on the frame rate and vice versa (*p* < 0.001).

The blocks analyzing resolution and color have only two levels per factor so sphericity checking is not applicable. In this case, the null hypothesis can be rejected for single effects in the high content variation group (*p* < 0.001), but not for the interaction (*p* = 0.232). The hypothesis of equal means cannot be rejected for any of the effects in the low variation group either (*p* = 0.246 for color, *p* = 1.131 for resolution and *p* = 0.841 for interaction).

Summarizing, the analysis of variance verifies that there is enough statistical significance to consider that the differences in the MOS values are due to differences in the bitrates and frame rates, but a similar conclusion for color/resolution can only be drawn for the high variation content block. For the color/resolution block with low variation content the results are inconclusive.

The data gathered about scientific utility has been used to compute a linear regression model between this measure and the MOS. Utility is mapped to integer values from 0 (useless) to 4 (very useful). The mean utilities for each value in the MOS scale have been computed and used as a dependent variable. The estimated regression line shown in Equation (3) has been obtained as a result:

(3) SU=0.8583 MOS−0.2409; 1<MOS<5

**Figure 6:**
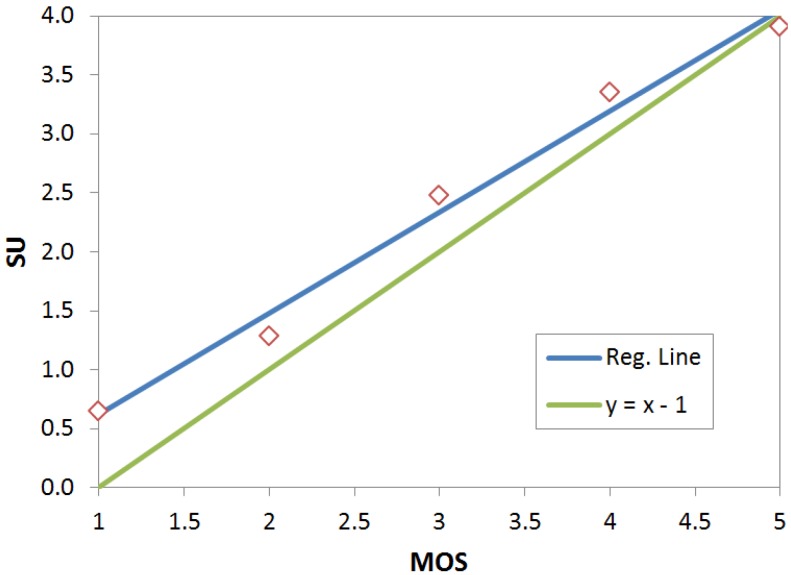
Scatter diagram for MOS *versus* scientific utility and estimated regression line.

Figure 6 plots the measured points, the regression line and equation y = x – 1. This equation represents the mapping function if there were an identity correspondence between MOS values and utility. It can be seen that though both lines are very close, the regression line is always above the identity line. This means that evaluators perceived a better utility than quality for all samples. This gap is wider, up to half a point, for low MOS and it decreases as the MOS increases.

The qualitative opinions gathered in the interviews cannot be analyzed through statistical methods, but are very consistent with the quantitative results. Most viewers stated that they preferred sharp images even if the video smoothness was not high. Another frequent remark was that grayscale images were as useful as the RGB videos for typical tasks. Also participants often mentioned that, in spite of their low definition, images are still useful for common processes such as identification and counting of species or identification of seafloor morphology.

The MOS results provided in this section show how analyzed video services achieve moderate quality scores. Even with the strong limitations in the encoding parameters, viewers in the test scored a good number of evaluation conditions in the fair category with some of them actually reaching the good category. These results notably differ from the G.1070 estimations for similar conditions. They allow planning of underwater video services with bitrates as low as 8 kbps at the application layer with an expected perceived quality of 3 out of 5 in the MOS scale and GOB between 20%–40%. When asked about the scientific utility of the samples, most of the scores fell in the moderately useful and quite useful categories. Finally, the viewers’ qualitative comments corroborate the quantitative analysis. This agreement supports the idea that MOS is being shifted because of the special access conditions.

## 4. Conclusions

Quality assessment is an important aspect of video service provisioning, but it turns out to be critical in highly constrained environments. It allows identifying the configuration parameters which make the difference between a useless service and a valuable one. This is the case for prospective underwater networks with a low available data rate, but also with the promising possibility of dramatically reducing costs of collecting images for scientific purposes.

Due to the problems associated with objective methods, this paper addresses the quality assessment issue from a subjective point of view. We have designed and performed an experiment under the guidelines of the ITU P.910 recommendation to gather quality data. The Spanish Institute of Oceanography has provided video sources from real underwater footage and a group of ocean scientist as evaluators.

The perceptual information (spatial and temporal) analysis of the video material is the first one, to the best of the authors’ knowledge, for underwater content. Most of the previous studies were focused on more general broadcasting contents.

The statistical processing of the collected data shows how the potential users of the video service rate conditions under test with grades between poor and good. A good number of conditions fall around the fair quality category, which supports the utility of this kind of video transmissions. Viewers often preferred 1 fps, grayscale sequences which obtained MOS > 3 (fair) with only one exception. 

Although considered higher bitrates also tended to produce better opinion values, these differences can be considered a marginal enhancement depending on the frame rates being compared. This can be seen for the high variation content samples with 1 fps, where there is an improvement of only 16% when bitrate is increased by 150%. Furthermore, MOS values are considerably higher than those predicted by the G.1070 parametric model for similar video conditions. 

These results confirm the initial hypothesis, and would justify a revision of the model in G.1070, which could benefit from taking into account additional factors. The results also lead to a better understating of video quality perception in underwater environments and could help to harness the existing technology to provide an effective instrument for oceanic research.
